# MR Imaging of Cochlear Modiolus and Endolymphatic Hydrops in Patients With Menière's Disease

**DOI:** 10.3389/fsurg.2021.667248

**Published:** 2021-07-20

**Authors:** Rita Sousa, Carla Guerreiro, Tiago Eça, Jorge Campos, Leonel Luis

**Affiliations:** ^1^Neuroradiology Department, Centro Hospitalar Universitário Lisboa Norte, Lisbon, Portugal; ^2^Otorhinolaryngology Department, Centro Hospitalar Universitário Lisboa Norte, Lisbon, Portugal; ^3^Imaging Department, Red Cross Hospital, Lisbon, Portugal; ^4^Clinical Physiology Translational Unit, Institute of Molecular Medicine, University of Lisbon, Lisbon, Portugal

**Keywords:** Menière's disease, endolymphatic hydrops, cochlear modiolus, magnetic resonance inner ear, membranous labyrinth

## Abstract

**Background:** Menière's disease (MD) is an inner ear disorder characterized by recurrent episodes of spontaneous vertigo, unilateral low-frequency sensorineural hearing loss, tinnitus, and aural fullness. Current diagnosis still often has to rely on subjective and audiometric criteria only, although endolymphatic hydrops is recognized as the pathophysiological substrate of the disease, having been demonstrated in anatomical pathological studies and by magnetic resonance (MRI). The modiolus has a close functional and anatomical relationship with the cochlear nerve and membranous labyrinth and can be evaluated with MRI but no data exist on the modiolar size in MD.

**Purpose:** Our purpose is to examine the following hypothesis. Is cochlear modiolus smaller in symptomatic ears in MD?

**Methods:** We used a retrospective 3 Tesla MR study (heavily T2-weighted 3D fast asymmetric spin-echo images and 0.5 mm slice thickness) comparing the mean modiolar area (MMA) in the index and best ears of eight patients with definite MD based on audiometric data. The obtained MMA values were compared against the audiometric data and the presence of vestibular endolymphatic hydrops.

**Results:** No differences were seen in MMA between best and worst ears. Ears with a pure tone average (PTA) ≥25 dB and more pronounced endolymphatic hydrops showed lower MMA (not statistically significant). Two patients with extreme endolymphatic hydrops showed a noteworthy ipsilateral decrease in the cochlear modiolus area.

**Conclusion:** No differences were seen in MMA between best and worst ears in definite MD. Worse hearing function (PTA ≥ 25dB) and more pronounced endolymphatic hydrops seem to be associated with lower MMA. This might be related to bone remodeling as a consequence of endolymphatic hydrops. Further research is needed to corroborate and explore these findings.

## Introduction

MD is a chronic disease with a prevalence of 200–500 per 100,000 individuals (1), characterized by a recurrent clinical syndrome of audiovestibular symptoms, namely spontaneous vertigo, unilateral hearing loss, aural fullness, and tinnitus (2).

Prosper Ménière, in 1861, was the first to recognize the inner ear at the origin of the symptoms (3), with endolymphatic hydrops only later, in 1937, being described by British (4) and Japanese (5) researchers.

Nowadays its cause remains undetermined, but the pathophysiological substrate seems to be the increase in the endolymphatic space of the membranous labyrinth, partially occupying the usual space of the perilymph. The molecular mechanism of the endolymphatic hydrops is still unknown, although genetic and inflammatory factors have been referred (6). ELH has been known to be the pathological basis of various pathophysiological changes of inner ear function (7).

Nowadays, endolymphatic hydrops can be demonstrated with MR (8), but current diagnostic criteria still remain symptom based and do not consider vestibular evaluation nor demonstration of endolymphatic hydrops (9), although this has been debated controversially (2).

Given the audiometric deterioration in the disease and the close relation of the modiolus with the endolymphatic space, a change in the structure of cochlear modiolus might be expected at least in symptomatic ears of patients with MD.

Some theories refer to an inflammatory mediated process in endolymphatic structures with micro ruptures of the membranous labyrinth, causing a sudden mixture between the perilymph and endolymph, resulting in physical and chemical changes in the cochlear and vestibular system (10, 11). This chronic process could result in a reactive bone process of hyperostosis and an increase of cochlear modiolus size. On the other side, a decrease of cochlear modiolus size could be explained by bone remodeling as a result of chronic increased pressure caused by enlarged endolymphatic space, at least in long-term disease or in cases with more pronounced endolymphatic hydrops.

Because we could not find sufficient data in relation to this question in the literature and clarify these controversial hypotheses, we decided to investigate the size of cochlear modiolus in 16 ears of eight patients with clinical definite MD.

## Materials and Methods

All studies were performed in a 3 Tesla MRI scanner (Philips Achieva–Philips Medical Systems, Best, Netherlands), using an 8-channel head coil. Thin-section heavily T2-weighted 3D fast spin-echo, TR 1500/TE 200, echo train length 61, field of view 150, slice thickness 0.5 mm, axial slab matrix 256, voxel size 0.5 AP/0.7 RL/0.5 FH and scan time 4.34 min.

Thin section MR images of 16 ears of eight patients with definite MD were obtained. Patients were all recruited from the outpatient otoneurological department of Hospital Santa Maria (Lisbon). They were clinically evaluated by one senior (LL) and one junior (TE) otoneurologist who classified the patients with definite MD according to the clinical Barany Society criteria (8) and defined the “symptomatic” (or “index”) and “asymptomatic” (“best”) ears according to clinical/audiometric evaluation. In each patient, the “index ear” was considered the one with a higher pure tone average (at 500 dB, 1,000 dB, 2,000 dB, and 4,000 dB), similar to the recently published concept of the “index ear” for MD (12).

In our study, we used a volumetric T2 acquisition with 0.5 mm thickness. Two neuroradiologists, blinded to the clinical diagnosis, independently evaluated the area of the cochlear modiolus on the MR console, using multiplanar reformatting. This area was measured in the axial slice in which the cochlear modiolus was visualized at its maximum size from the thin section T2-weighted images (midmodiolar level). A region of interest was manually drawn and measured by each radiologist twice: the measured area outlined the low signal trapezoidal or triangular shape at the axis of the basal turn of the cochlea, or the middle and basal turn, excluding the thin free part of the osseous spiral lamina and interscalar septum (13, 14). The average of the two measurements was obtained for each radiologist and the final value was obtained by averaging the values obtained by each radiologist.

Additionally, the vestibular endolymphatic space was evaluated, based on MR images obtained with HYDROPS protocol 4 h after intravenous injection of a single dose of gadolinium, as previously described (15).

The area of vestibular endolymphatic space and the area of the total membranous labyrinth was manually traced by a radiologist, using multiplanar reformatting, according to the plane of the lateral semicircular canal (including the ampulla in the measurement), using volumetric acquisitions (the volumetric HYDROPS and T2, respectively). The final value was obtained as the percentage of endolymphatic area to total labyrinth area. We couldn't quantify cochlear endolymphatic space because of image quality limitations. All image evaluation procedures were performed by researchers blinded to the clinical data of the patients.

The two groups were compared using the Mann-Whitney *U* test. A *p*-value of 0.05 was considered statistically significant. Interrater reliability was also assessed using the intraclass correlation coefficient (ICC).

## Results

Cochlear modiolus area, PTA, and vestibular endolymphatic space were registered in Table 1 for “index” and “best” ears. We tried to avoid the terms symptomatic and asymptomatic because some “asymptomatic” ears are not truly healthy and some show elevated PTA (higher than 25dB) (patients two, six, seven, and eight). This is explained by the tendency of bilateral affection in the disease (16, 17).

**Table 1:** PTA, mean modiolus area, vestibular endolymphatic hydrops, and disease duration of worst and best ears of eight patients with clinical criteria of definite MD.

	**Ear group**	**PTA (dB)**	**Mean modiolus area (mm^**2**^)**	**Vestibular endolymphatic hydrops (%)**	**Disease duration (years)**	**Number of vertigo attacks (nr/year)**	**Duration of vertigo attacks**	**Fluctuating symptoms**	**Instability between attacks**
Patient 1	Index	55	3.10	82	3	6	20 min−12 h	Hypoacusia Tinnitus	No
	Best	15	3.53	66					
Patient 2	Index	69	3.69	86	1	10	20 min−12 h	Hypoacusia Aural fullness Tinnitus	No
	Best	28	3.93	50					
Patient 3	Index	55	3.94	63	6	3	20 min−12 h	Hypoacusia Aural fullness Tinnitus	No
	Best	7	3.76	61					
Patient 4	Index	41	3.82	47	5	5	20 min−12 h	Tinnitus Aural fullness	Yes
	Best	13	3.76	57					
Patient 5	Index	41	3.59	21	2	3	20 min-12 h	Hypoacusia Aural fullness Tinnitus	No
	Best	13	3.46	29					
Patient 6	Index	59	3.46	70	2	2	20 min−12 h	Hypoacusia Aural fullness Tinnitus	No
	Best	53	3.34	58					
Patient 7	Index	61	3.21	50	24	5	20 min−12 h	Hypoacusia Aural fullness Tinnitus	Yes
	Best	30	3.36	35					
Patient 8	Index	39	3.03	28	13	3	20 min−12 h	Tinnitus Aural fullness	No
	Best	28	3.28	57					

Three MD patients were male and five were female. Ages ranged from 47 to 52 in MD group (mean age of 48). Disease duration ranged from 2 to 24 years (mean duration of 7 years). All patients had spontaneous vertigo attacks lasting from 20 min to 12 h and fluctuating symptoms; number of vertigo attacks ranged from 2 to 10 episodes/year; and postural instability between attacks was described by two patients (Table 1). Pure tone average audiometry (PTA) ranged from 15 to 55 dB (mean PTA of 44dB).

**Figure 1:**
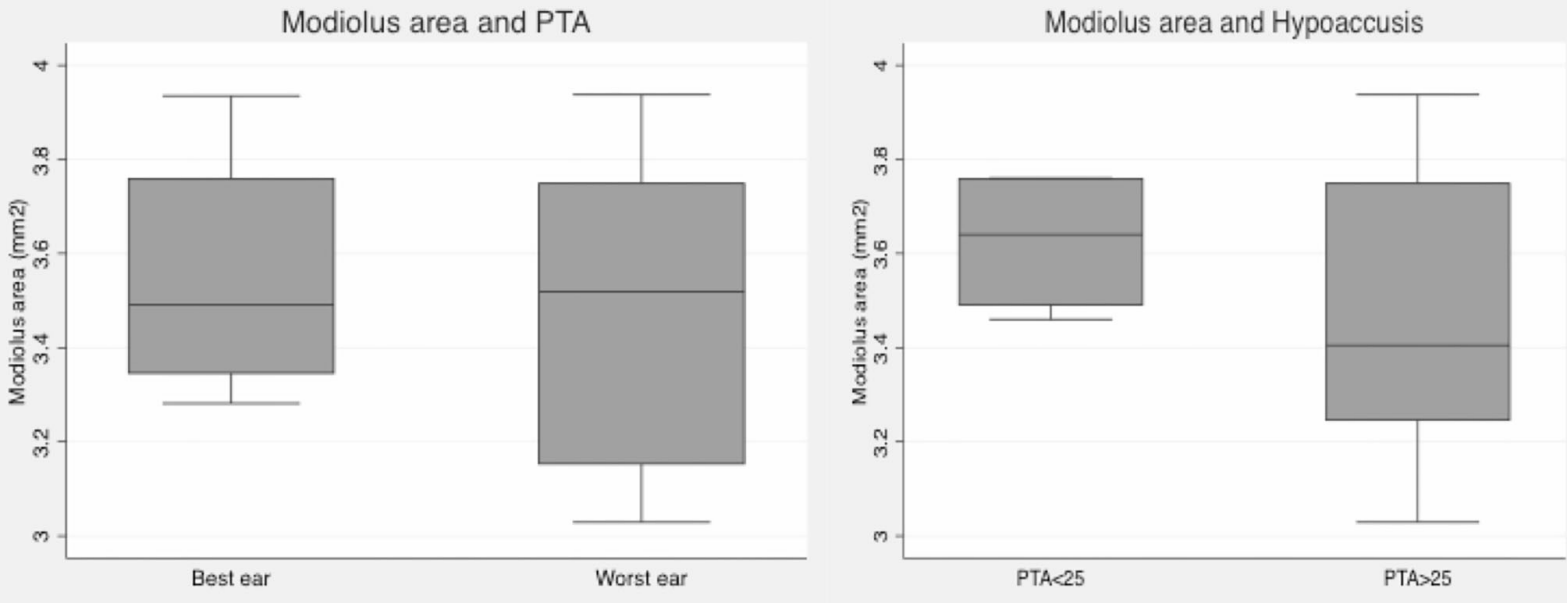
Box plot graphics. Left: Mean modiolar area in best and index ears (based on PTA): no differences were found. Right: Mean modiolar area in ears with normal PTA (<25dB) and with hearing loss (≥25dB): ears with PTA≥25 have smaller modiolus areas, but differences were not statistically significant.

The MMA in this MD population was 3.52 mm^2^ (3.03 to 3.94 mm^2^). Interrater reliability was good: ICC = 0.90, with a 95% confidence interval (0.59–0.97). We did not find differences of MMA between “index ears” (3.51 mm^2^) and “best ears” groups (3.52 mm^2^) (Figure 1), but ears with PTA higher than 25dB had smaller modiolus areas, although this is not a statistically significant finding (*p* = 0.3320).

Vestibular endolymphatic hydrops was present in 88% of patients with definite MD (7/8 patients). Vestibular endolymphatic hydrops was found in 11/16 ears (69%) according to Barath's criteria (18) (cut-off of 50%) and 13/16 ears (81%) according to Nakashima's criteria (19) (cut-off of 33%).

**Figure 2:**
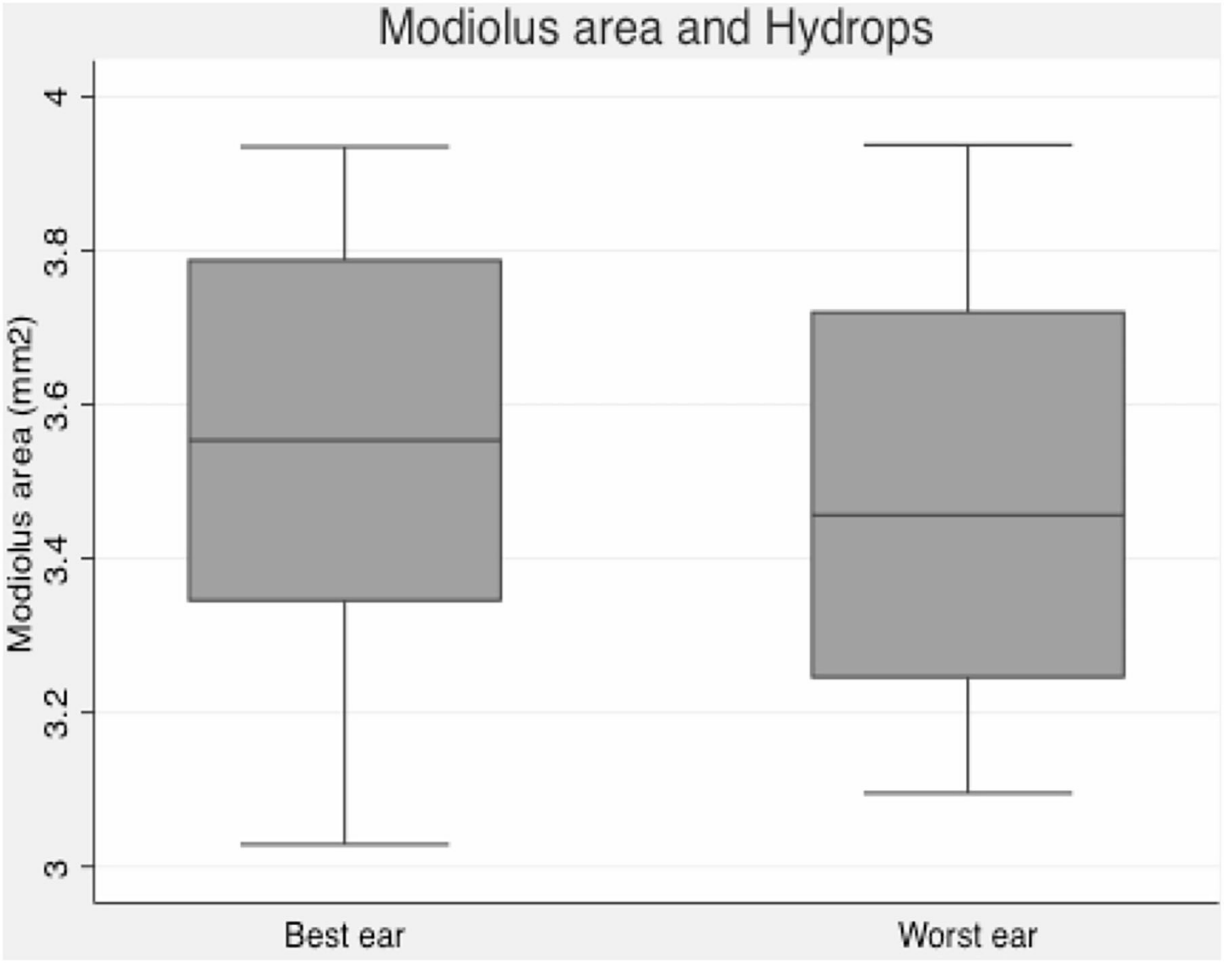
Box plot graphic. Modiolus area and endolymphatic hydrops–slightly smaller modiolus in ears with more pronounced vestibular hydrops.

Mean vestibular endolymphatic space was 56% in symptomatic ears and 52% in asymptomatic ears. We got slightly smaller modiolus in “index” endolymphatic hydrops ears, although this is not a statistically significant finding (Figure 2).

**Figure 3:**
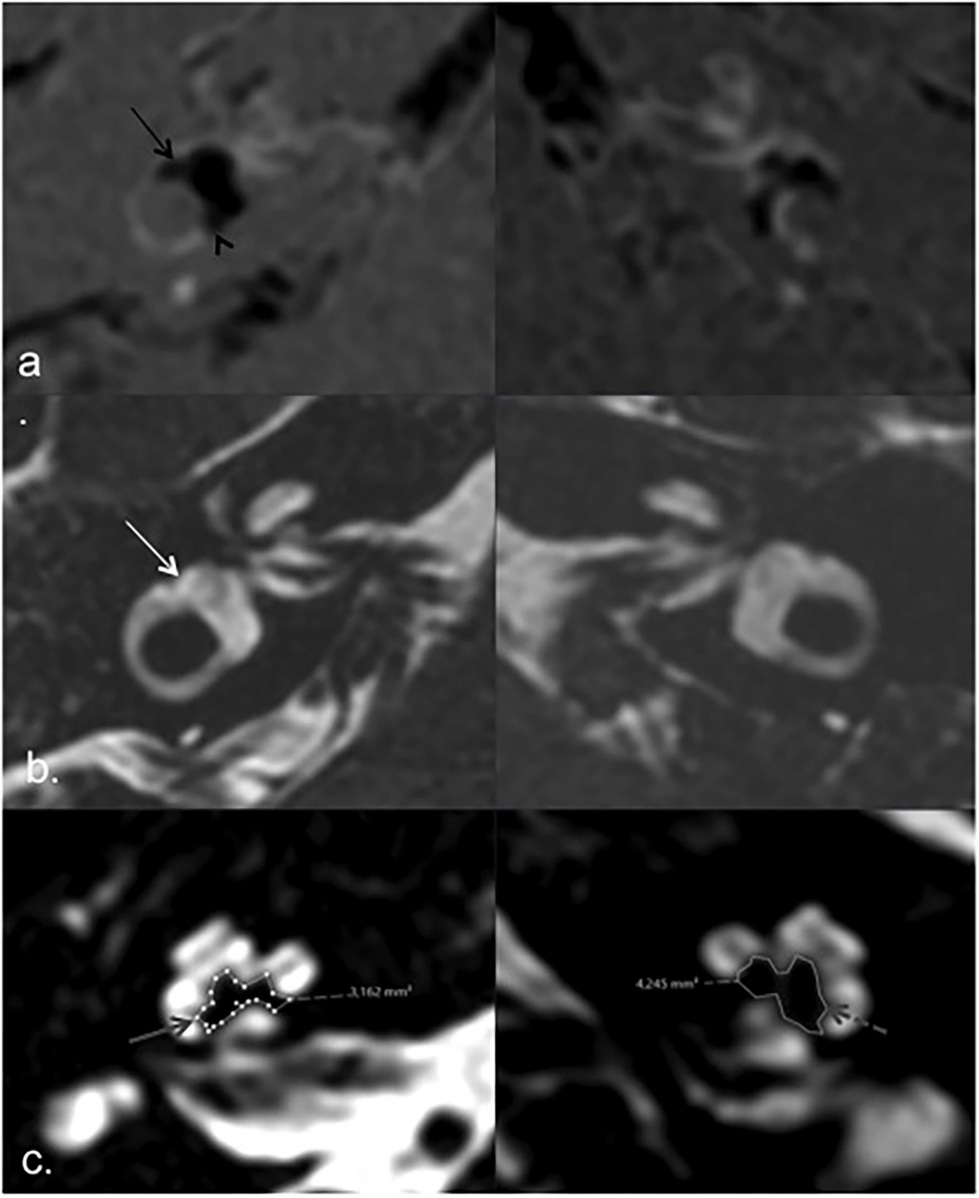
MRI of patient 2. HYDROPS **(a)** and T2 3D TSE **(b)** axial images according to lateral semicircular canal plane, showing endolymphatic (black arrows) and total vestibular labyrinth (white arrow) respectively. Extreme right vestibular endolymphatic hydrops with herniation of endolymph into de ampullary (black arrow) and non-ampullary (black arrowhead) endings of the lateral semicircular canal in right ear (index ear). Axial T2 image according to the greater axis of cochlear modiolus **(c)**, showing marked asymmetric modiolar areas, smaller on the right side (solid gray arrow) compared to the left asymptomatic side (dashed gray arrow).

**Figure 4:**
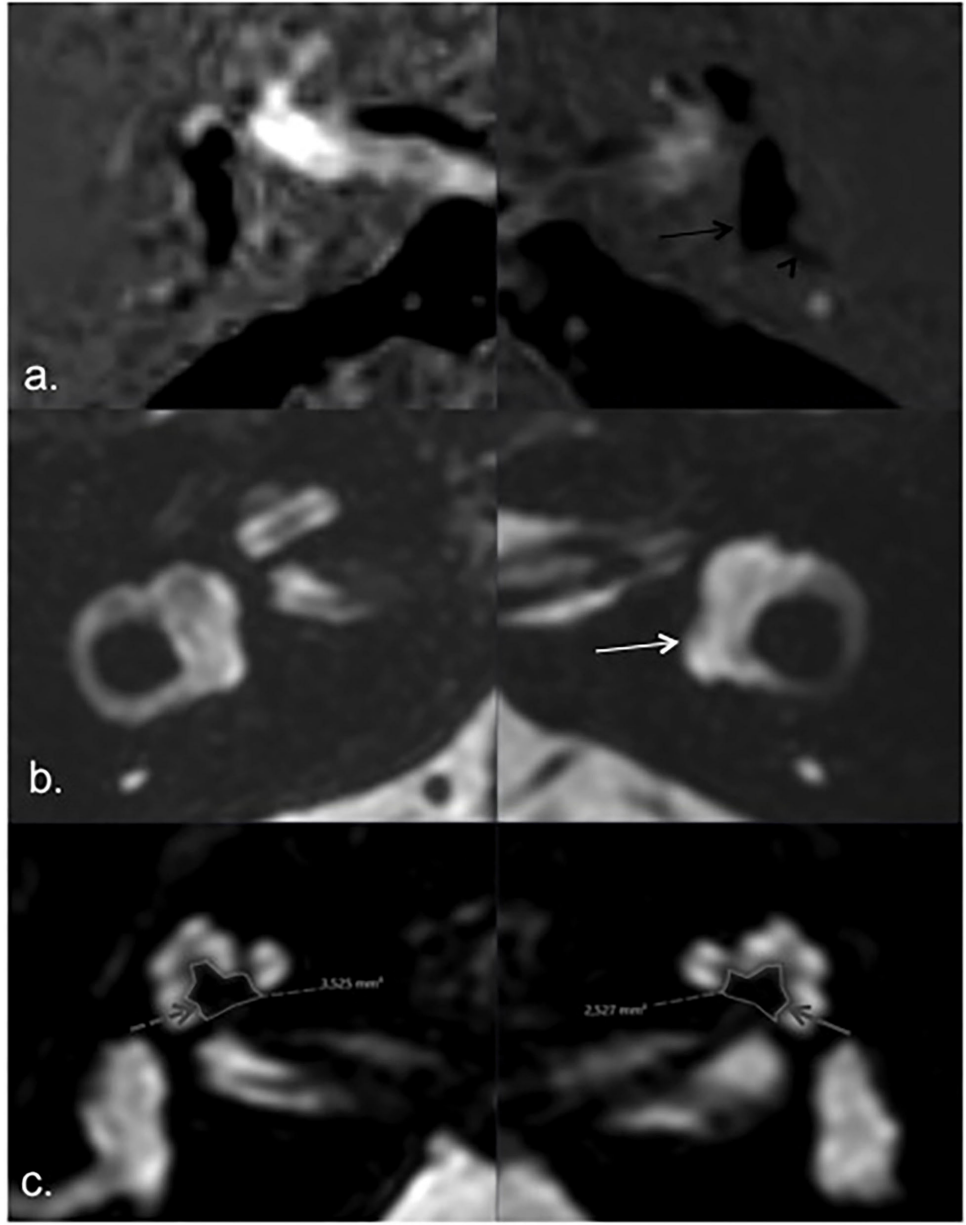
MRI of patient 1. HYDROPS **(a)** and T2 **(b)** axial images showing endolymphatic (black arrow) and total membranous labyrinth (white arrow) respectively; bilateral vestibular endolymphatic hydrops, more pronounced on the left side (a. black arrow) with herniation of the utricle into the non-ampullary limb of the lateral semicircular canal (black arrowhead). Axial T2 image according to the greater axis of cochlear modiolus **(c)**, showing marked asymmetric modiolar areas, smaller on the left side (index ear) (solid gray arrow) compared to the left asymptomatic side (dashed gray arrow).

When we analyzed PTA, modiolus area, and endolymphatic space for each individual patient, we found a consistently decreased modiolar area in the index ears in only three patients (patients one, two, and seven), but this was more evident in patients one and two, which interestingly had more extreme endolymphatic hydrops and a higher number of acute episodes and a relatively recent diagnosis of the disease (Figures 3, 4, Table 1). Patient seven had a longer disease duration and described persistent postural instability (Table 1).

## Discussion

According to previous histological studies (11), the cochlear modiolus has a base of 4 mm and a height of 3, which gives an area of 6 mm^2^ if we assume it has a triangular shape, which is not always true. Naganawa et al. first measured cochlear modiolus with MRI in 1999 in asymptomatic healthy volunteers (13); they used a 1.5 Tesla scanner, surface coils, matrix of 512, and 0.8 mm slice thickness, and their measurements showed areas ranging from 4.1 to 5.8 mm^2^ in healthy subjects, which is slightly smaller than previously documented. These values are in discrepancy with those previously obtained with CT (14) but they can be explained by limitations inherent to the CT technique. With CT one can only measure the calcified portion of modiolus, which can explain lower modiolar areas in some ears.

To our knowledge, no data exist regarding cochlear modiolar size in MD, although it has been studied in patients with large endolymphatic duct and sac (13). Naganawa et al. (13) found a decrease in the size of the modiolus in 8 of 12 patients with large endolymphatic sac and duct and modiolus >4 mm^2^ in 4 of 12 ears; all the 10 ears of healthy volunteers showed modiolus areas >4 mm^2^. These data suggested that hearing loss in patients with large endolymphatic duct and sac might be caused by microscopic changes that are not visible on MRI and do not support the theory that hearing loss was caused by hydrostatic subarachnoid pressure into the labyrinth through a deficient modiolus.

In our study we used a 3-tesla MR scanner and a thinner slice thickness (0.5 mm) (instead of 0.8 mm used by Naganawa) in order to decrease the partial volume effect. Naganawa et al. (13), however, used surface coils, a smaller field of view, and larger matrix size. The same method of measuring the maximum cross-sectional area of the cochlear modiolus was applied (13), excluding the osseous spiral lamina and interscalar septum. Similarly, two observers manually measured the areas twice and the mean value of all measurements was used for analysis. Although there is a substantial subjectivity inherent to human judgment in defining the region of interest, especially in defining the base of the triangle on MR, our interobserver agreement was good with this method.

A volumetric measurement should be more precise, but we would need higher-quality images. As this was a retrospective study, we did not have data from a group of healthy volunteers (it would be difficult to reproduce the same conditions in the new available MR scanner). For this reason, we can only compare our MD data with healthy volunteers data from previous histological (11, 20) and MR (14) data. Our data showed mean areas of 3.52 mm^2^ (3.03–3.94 mm^2^), which seems to be lower than in the healthy population. However, we cannot make definite conclusions regarding this and future prospective controlled studies can confirm this finding with greater confidence: we will expect a global decrease in modiolus size in index and non-index ears in MD compared to the healthy asymptomatic control group.

In this study, we did not find significant statistical differences in MMA between “asymptomatic” and index ears in MD. Maybe this can be related to the fact that the “asymptomatic” or “best” ear is not truly healthy in some patients as we can see from the PTA records. This explains why a slight decrease in MMA is seen in ears with PTA higher than 25 dB or in ears with larger endolymphatic spaces. This aspect was more evident in two particular patients (patients one and two) even in relatively short-term disease (3 and 1 years, respectively). Our endolymphatic space areas refer to the vestibular component only because of image quality limitations in cochlear endolymphatic space.

Increased hydrostatic pressure can explain these findings, based on increased endolymph fluid and decreased microvascular supply (stria vascularis), causing bone remodeling and demineralization, respectively. This aspect has been demonstrated in previous histological studies (11, 20) and should also be corroborated in future MR investigations with a larger sample of patients with MD, or even better, with a larger sample of patients with hydropic ear disease. We think a study that aims to compare MMA between ears with and without endolymphatic hydrops (not only MD) can show significant differences, as the presumed underlying mechanism of modiolar decrease is present (bone remodeling), independently of the (subjective) clinical diagnosis. However, this might be observed mainly in more pronounced endolymphatic hydrops cases or in long-standing ones. We emphasize that the time and duration of the disease were determined based on the time of the disease from the definitive diagnosis. In both cases, the patients presented with audiovestibular symptoms 6 and 5 years before the definitive diagnosis (although the diagnosis of definite MD is relatively recent).

In fact, it seems MD is an entity whose clinical criteria have some inherent diagnostic subjectivity based on symptoms only, and MD seems to include a wide range of distinct etiologies, being difficult to unify in a single entity. Endolymphatic hydrops seems to be present in most patients with a clinical diagnosis but also in other patients with an incomplete clinical picture of MD or secondary to other etiologies (congenital, tumors, trauma, among others), which has lead to the concept of Hydropic Ear Disease, encompassing all typical and atypical variants of MD in one logical framework (21–23). For these reasons, in further studies, it should be interesting to study this particular subject of cochlear modiolus size in a population with documented hydropic ear (21–23), as suggested by our two cases of extreme hydrops and decreased modiolar size.

## Conclusions

In the last years, a lot has been published regarding imaging in MD, mainly related to endolymphatic hydrops, which is the pathophysiological correlate of the disease. Endolymphatic hydrops is present in the greater majority of MD patients but can be also be found in other entities. Recently other additional imaging signs have been shown to increase the specificity of endolymphatic hydrops in MD (besides the diagnosis remains clinical only), such as the increased intensity of the perilymphatic space after gadolinium injection in the index ear (24), which represents an increase in the permeability of the hemato-perilymphatic barrier. In this study, we focused on another still unexplored finding: cochlear modiolus size and its relationship with endolymphatic hydrops.

The modiolus area is not significantly different between the index and best ears in MD, which can be explained by the tendency of bilateral affection of the disease and might reflect its systemic nature. However, the modiolus area seems to be smaller in ears with extreme endolymphatic hydrops, causing worse hearing function in patients with clinical criteria for definite MD, which can be justified by increased hydrostatic pressure resulting in bone remodeling of cochlear modiolus and cochlear nerve endings damage. MR imaging of the cochlear modiolus deserves more investigation, however, especially in patients with endolymphatic hydrops, with or without fulfillment of traditional clinical criteria for MD (termed “hydropic disease”) (21, 22). Assessment of the modiolus area may be a useful evaluation finding, easy to assess, with a high inter-observer agreement, which favors its applicability in clinical practice. Although this study has limitations inherent to its retrospective design, such as a reduced number of patients and the absence of a healthy control group, it raises new clues regarding imaging in MD/ hydropic disease that have been previously unreported. Further controlled and prospective studies, with a larger number of patients, are needed to corroborate and explore these findings.

## Data Availability Statement

The original contributions presented in the study are included in the article/supplementary material, further inquiries can be directed to the corresponding author.

## Ethics Statement

The studies involving human participants were reviewed and approved by Comissão de Ética da Faculdade de Medicina da Universidade de Lisboa. The patients/participants provided their written informed consent to participate in this study.

## Author Contributions

RS, CG, TE, JC, and LL contributed to conception and design of the study. RS and CG evaluated the MR images. TE and LL made the clinical evaluation. RS and CG organized the database. CG performed the statistical analysis. RS wrote the first draft of the manuscript. RS, CG, and TE wrote sections of the manuscript. All authors contributed to manuscript revision, read, and approved the submitted version.

## Conflict of Interest

The authors declare that the research was conducted in the absence of any commercial or financial relationships that could be construed as a potential conflict of interest.
